# Hsa_circRNA_0101125 Promotes Proliferation, Migration, and Invasion of Esophageal Squamous Cell Carcinoma via miR‐143‐3p/ZNF148 Axis

**DOI:** 10.1096/fj.202500257R

**Published:** 2025-09-29

**Authors:** Hao Chen, Songtao Xue, Shiqiang Cao, Jiarong Zhang, Yuanpu Wei, Chun Chen, Guobing Xu

**Affiliations:** ^1^ Department of Thoracic Surgery Fujian Medical University Union Hospital Fuzhou city Fujian province China; ^2^ Key Laboratory of Cardio‐Thoracic Surgery (Fujian Medical University) Fujian Province University Fuzhou city Fujian province China; ^3^ Clinical Research Center for Thoracic Tumors of Fujian Province Fuzhou city Fujian province China; ^4^ National Key Clinical Specialty of Thoracic Surgery Fuzhou city Fujian province China

**Keywords:** epithelial‐mesenchymal transition, esophageal cancer, hsa_circRNA_0101125, miR‐143‐3p, ZNF148

## Abstract

Esophageal squamous cell carcinoma (ESCC) is a highly invasive and metastatic malignancy. Circular RNAs (circRNAs), including hsa_circRNA_0101125, have been implicated in regulating cancer metastasis. But its potential roles in ESCC remain underexplored, particularly in relation to miR‐143‐3p. This study aimed to investigate the role of hsa_circRNA_0101125/miR‐143‐3p in the occurrence and development of ESCC. KYSE150 and ECA109 cells were used as in vitro models to evaluate the biological effects of hsa_circRNA_0101125. The interaction between ZNF148 and miR‐143‐3p was confirmed by dual‐luciferase assay. To assess the functional role of this pathway, cells were transfected with sh‐hsa_circRNA_0101125 vectors and/or miR‐143‐3p inhibitors, followed by assays for cell viability (CCK‐8), migration (wound healing assay), and invasion (Transwell assay). Transcriptions and expressions of hsa_circRNA_0101125, miR‐143‐3p, and ZNF148 were measured by RT‐qPCR and western blot. Results showed that hsa_circRNA_0101125 was localized in the cytoplasm and negatively regulated miR‐143‐3p, which targeted ZNF148. Inhibition of hsa_circRNA_0101125 significantly reduced cell viability, migration, and invasion, downregulated ZNF148 and Vimentin, and upregulated miR‐143‐3p and E‐cadherin expressions. In contrast, downregulating miR‐143‐3p abolished these effects. Therefore, hsa_circRNA_0101125 promotes the proliferation, migration, and invasion of ESCC cells by inhibiting the expression of miR‐143‐3p.

## Graphical abstract

hsa_circRNA_0101125 promotes esophageal squamous cell carcinoma progression by sponging miR‐143‐3p and upregulating ZNF148 expression. This regulatory axis enhances tumor cell proliferation, migration, and invasion, likely by modulating EMT‐related markers including E‐cadherin and Vimentin. Inhibiting hsa_circRNA_0101125/miR‐143‐3p/ZNF148 might ameliorate esophageal squamous cell carcinoma progression. The hsa_circRNA_0101125/miR‐143‐3p/ZNF148 axis may serve as a potential therapeutic target in ESCC.

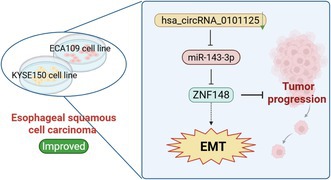

**Abbreviations**
circRNA: Circular RNA
CSCs: Cancer Stem Cells
EMT: Epithelial‐Mesenchymal Transition
miR: MicroRNA
ZEB1: Zinc Finger E‐box Binding Homeobox 1
ZHX3: Zinc Fingers and Homeoboxes 3
ZNF148: Zinc Finger Protein 148

## 1 Introduction

Esophageal cancer is an invasive malignant tumor that causes over 400,000 deaths worldwide every year [1]. There are two main subtypes of esophageal cancer with different epidemiological distributions and pathogenesis, namely esophageal squamous cell carcinoma (ESCC) and adenocarcinoma (EAC) [2]. ESCC accounts for over 90% of esophageal cancer [3]. Preoperative radiotherapy is an important method for treating ESCC in clinical practice. Due to the invasive and metastatic ability of ESCC cells, the prognosis of ESCC is poor, and the survival rate after initial treatment is less than 13% [2, 3, 4]. Exploring the cellular function of ESCC cells and how to inhibit the proliferation, invasion, and metastasis of cancer cells is of great significance for the treatment of ESCC.

Circular RNAs (circRNAs) are a class of non‐coding RNAs that lack a 5′ end cap and a 3′ end poly (A) tail, and form ring structures by covalent bonds. CircRNAs can serve as miRNA sponges (competing endogenous RNA, ceRNA mechanism) to regulate gene expression in a variety of cancers, thereby affecting the proliferation, migration, and invasion of cancer cells [5]. CircRNAs also play important regulatory roles in ESCC. For example, circRNA_100367 can regulate the radiosensitivity of ESCC through the miR‐217/Wnt3 pathway [6]. In our previous study, we identified hsa_circ_0101125 as significantly upregulated in ESCC tissues by circRNA microarray and RT‐qPCR analysis [7]. Therefore, we speculate that hsa_circRNA_0101125 may be one of the regulatory genes involved in the occurrence of ESCC.

MicroRNAs (miRs) are non‐coding single‐stranded RNAs that contain 20–23 nucleotides, which block mRNA translation and regulate target gene expression by degrading mRNA or by complementing mRNA coding sequences [8]. In the ceRNA mechanism, circRNA acts as a ceRNA that competes with mRNA to bind to the same miRNA. CircRNA can inhibit miRNA expression and relieve the inhibitory effect of miRNA on its target gene [9, 10]. MiR‐143‐3p is a tumor suppressor that can inhibit the invasion and migration of ESCC cells [11, 12]. There have been no reports on the relevant mechanisms underlying the role of hsa_circRNA_0101125 in regulating cancer cells until now. Further exploring whether hsa_circRNA_0101125 affects the proliferation, migration, and invasion of ESCC cells by regulating miR‐143‐3p is of great importance.

Zinc finger proteins (ZFPs) represent one of the largest families of transcription factors and are deeply involved in diverse cancer‐related processes, including proliferation, apoptosis, angiogenesis, and metastasis [13, 14]. Zinc Finger E‐box Binding Homeobox 1 (ZEB1) and zinc fingers and homeoboxes 3 (ZHX3) have been extensively implicated in promoting epithelial‐mesenchymal transition (EMT), enhancing tumor cell migration and invasion, and thereby contributing to cancer aggressiveness and poor prognosis [15, 16, 17, 18]. Especially, Zinc Finger Protein 148 (ZNF148) expression is significantly elevated in ESCC tumor tissues compared to adjacent normal mucosa. High ZNF148 expression is closely correlated with advanced TNM stage, lymph node metastasis, and poorer overall survival. Multivariate Cox regression analysis confirmed that ZNF148 is an independent prognostic marker, suggesting that it may facilitate tumor progression and serve as a valuable biomarker for predicting clinical outcomes in ESCC patients [19]. Moreover, while ZEB1 was identified as a direct downstream target of miR‐143‐3p [20], the regulatory relationships involving ZHX3 and ZNF148 in ESCC remain to be elucidated given their structural similarity and potential roles in cancer progression.

Therefore, this study aimed to elucidate the regulatory role of the hsa_circRNA_0101125/miR‐143‐3p/ZNF148 axis in ESCC progression. To achieve this, we employed two representative ESCC cell lines, KYSE150 and ECA109, and conducted a series of functional experiments, including in situ hybridization, RT‐qPCR, dual‐luciferase reporter assays, and phenotypic analyses of cell proliferation, migration, and invasion. The results demonstrated that hsa_circRNA_0101125 acts as a molecular sponge for miR‐143‐3p, thereby enhancing ZNF148 expression and promoting ESCC cell proliferation and motility. These findings might reveal a novel circRNA‐mediated ceRNA regulatory axis and suggest that hsa_circRNA_0101125 may serve as a potential biomarker and therapeutic target in ESCC.

## 2 Materials and Methods

### 2.1 In Situ Hybridization

The fluorescence in situ hybridization (FISH) method was used to detect the localization of hsa_circRNA_0101125 in cultured ESCC cells. Cells were seeded onto sterile glass coverslips placed in 24‐well plates and cultured under standard conditions to 70%–80% confluence. These coverslips served as cell climbing slides for subsequent fluorescence in situ FISH. After paraffin removal and hydration, the slices were treated with pepsin diluted with 3% citric acid. In situ hybridization was performed using the hsa_circRNA_0101125 in situ hybridization kit (MK11075‐h, Boster, Wuhan, China), followed by fluorescence staining with Cy3 fluorescent antibody (Boster, Wuhan, China). Finally, the nuclei were stained with DAPI and the slices were observed under a fluorescence microscope (CKX53, Olympus, Tokyo, Japan).

### 2.2 Cell Culture and Cell Transfection

Esophageal cancer cell lines KYSE‐150 (CL‐0737, Procell, Wuhan, China) and ECA‐109 (BNCC342591, BeNa Culture Collection, Beijing, China) were cultured in complete culture medium RPMI‐1640 (KGM31800S, KeyGen Biotech, China) containing 2 μg/mL puromycin (P8230, Solarbio, Beijing, China) and 10% fetal bovine serum (FBS; Gibco, New York, USA) at 37°C in a culture incubator containing 5% CO_2_ and 95% air.

To investigate the functions of hsa_circRNA_0101125 and miR‐143‐3p in ESCC cells, the CH‐plvx‐shRNA‐hsa_circRNA_0101125 vector (synthesized by General Biol (Anhui) Co. LTD, Anhui, China), miR‐143‐3p mimic, and miR‐143‐3p inhibitor (both synthesized by General Biol (Anhui) Co. LTD, Anhui, China) were transfected into the cells using Lipofectamine 3000 (L3000001, Thermo Fisher, Massachusetts, USA) according to the manufacturer's protocol. The working concentration for both the miRNA mimic and inhibitor was 25 nM, as recommended in the product datasheet. After transfection, cells were cultured for an additional 48 h before subsequent analyses. The mimics and inhibitors used in this study were shown in Table 1.

**TABLE 1:** Mimics and inhibitors used in this study.

Name	Sequences
miR‐143‐3p mimic	sense 5′‐UGAGAUGAAGCACUGUAGCUC‐3′ anti‐sense 5′‐GAGCUACAGUGCUUCAUCUCA‐3′
mimic NC	sense 5′‐UCACAACCUCCUAGAAAGAGUAGA‐3′ anti‐sense 5′‐UCUACUCUUUCUAGGAGGUUGUGA‐3′
miR‐143‐3p inhibitor	5′‐GAGCUACAGUGCUUCAUCUCA‐3′
inhibitor NC	5′‐UCUACUCUUUCUAGGAGGUUGUGA‐3′

Abbreviation: MiR, microRNA.

### 2.3 CCK‐8 Assay

CCK‐8 Cell Proliferation Detection Kit (KGA317) was purchased from KeyGEN BioTECH (Jiangsu, China). A total of 1 × 10^4^ cells per well were seeded into 96‐well plates and cultured overnight to allow adherence. The culture medium was then replaced with 100 μL of fresh complete medium per well, followed by the addition of 10 μL of CCK‐8 reagent. Plates were incubated at 37°C for 2 h, and absorbance was measured at 450 nm using a microplate reader (WD‐2012B, Beijing Liuyi, China).

### 2.4 Wound Healing Assay

ESCC cells in good condition were seeded into a 24‐well plate at a density of 1 × 10^6^ cells/well and cultured overnight to ensure full adherence. Cells in good condition were defined as those displaying firm adherence, clear cytoplasm, and intact cell membranes under inverted microscopy. A straight scratch was made across the cell monolayer using a sterile 200 μL pipette tip. Detached cells were removed by gently washing with PBS, and serum‐free medium was added to minimize proliferation. The initial wound width (0 h) was recorded under an inverted microscope (BX43, Olympus, Tokyo, Japan). After 24 h and 48 h of incubation at 37°C, the same wound area was imaged again. The wound closure rate was calculated using ImageJ software (NIH, USA) as: Wound healing (%) = [(Width_24h/48h_—Width_0h_)/Width_0h_] × 100%. Each experiment was performed in triplicate.

### 2.5 Transwell Invasion Assay

Transwell chambers with 8 μm pore size were pre‐coated with 60 μL of diluted Matrigel (1:6 in serum‐free medium; 356 234, BD Biosciences, New Jersey, USA) and incubated at 37°C for 1 h. Cells in good condition were defined as those displaying firm adherence, clear cytoplasm, and intact cell membranes under inverted microscopy. ESCC cells in good condition were digested, washed with PBS, and resuspended in serum‐free medium. A total of 200 μL of the cell suspension (1 × 10^5^ cells/well) was seeded into the upper chamber, and 600 μL of complete medium containing 10% FBS was added to the lower chamber as a chemoattractant. The invaded cells on the underside of the membrane were fixed with 4% paraformaldehyde for 20 min and stained with 0.1% crystal violet for 30 min. The cells in the small chamber were wiped off with a cotton swab and observed under a microscope (BX43, Olympius, Tokyo, Japan). After taking photos, the pictures taken are quantified using the Image J software. For semi‐quantitative measurement, crystal violet was eluted with 33% acetic acid for 10 min, and the absorbance was measured at 562 nm using a microplate reader (WD‐2012B, Liuyi Instrument, China). Experiments were conducted in triplicate.

### 2.6 Construction of Dual‐Luciferase Reporter Vectors

The wild‐type (WT) and mutant (MUT) 3′ untranslated region (3′UTR) sequences of ZNF148 containing the predicted miR‐143‐3p binding site were synthesized and cloned into the pmirGLO dual‐luciferase vector (Promega, Madison, WI, USA) using SacI and XhoI restriction sites. The predicted binding site of miR‐143‐3p was 5′‐UGAGAUGAAGCACUGUAGCUC‐3′.

The WT‐ZNF148 3′UTR sequence included the intact miR‐143‐3p binding site, while in the MUT‐ZNF148 construct, several nucleotides at the binding site were substituted to disrupt miR‐143‐3p pairing. Plasmids were transformed into DH5α competent cells selected in LB medium containing 100 μg/mL ampicillin. The plasmid was extracted and RT‐qPCR was performed for validation. The sequences of WT‐ZNF148 (Wild Type‐ZNF148) were GAGCTCTTAGATTTTTAGGTTTGATATGGTTGTGTCATAATCAT CTCGTAATTGACAATTTTAACTTTTGGCAATAAAAGGAAATTGGGATATCTTTGGAACTGTAAAACCTGGTTTACTCGAG. The sequences of MUT‐ZNF148 (Mutant‐ZNF148) were GAGCTCTTAGATTTTTAGGTTTGATATGGTTGTGGACGAAGACGAGA GTAATTGACAATTTTAACTTTTGGCAATAAAAGGAAATTGGGATCGAGTTGGAACTGTAAAACCTGGTTTACTCGAG.

For the reporter assay, KYSE‐150 and ECA‐109 cells were co‐transfected with either WT‐ZNF148‐pmirGLO or MUT‐ZNF148‐pmirGLO vectors and miR‐143‐3p mimic or negative control mimic (25 nM) in 24‐well plates. After 48 h of transfection, luciferase activity was measured using the Dual‐Luciferase Reporter Assay System (E1910, Promega, Wisconsin, USA) according to the manufacturer's instructions. Firefly luciferase activity was normalized to Renilla luciferase activity.

### 2.7 Dual‐Luciferase Assay

KYSE150 and ECA109 cells were seeded in 24‐well plates and co‐transfected with either WT‐ZNF148‐pmirGLO or MUT‐ZNF148‐pmirGLO reporter plasmids (Promega, USA), together with 25 nM miR‐143‐3p mimic or negative control mimic, using Lipofectamine 3000 and P3000 reagent (L3000001, Thermo Fisher, Massachusetts, USA) according to the manufacturer's protocol. In brief, plasmids and mimics were diluted in Opti‐MEM medium and mixed with transfection reagents to form complexes. After incubation, the complexes were added to the cells and incubated for 48 h. Cells were then lysed on ice using lysis buffer; the lysed cell samples were stored at −80°C. Cell lysate from each group was taken and added into the 96‐well plate, and luciferase test reagent was added into each well to detect the activity of firefly luciferase. Then, Renilla luciferase test reagent (substrate: buffer =1:100) was added into each well, and the Renilla luciferase activity was detected. All experiments were performed in triplicate.

### 2.8 RT‐qPCR Detection

Total RNA was extracted from ESCC cells using the Trizol reagent (CW0580S, CWBIO, Jiangsu, China). RNA/miRNA was extracted using the RNA ultrapure extraction kit (CW0581M, CWBIO, Jiangsu, China)/miRNA ultrapure extraction kit (CW0627S, CWBIO, Jiangsu, China), and mRNA concentration and purity were measured using a UV visible spectrophotometer. For mRNA reverse transcription, the PrimeScriptTM RT reagent kit (RR047A; Takara Medical Technology, Beijing, China) was used to generate cDNA from the extracted RNA following the manufacturer's protocol. For miRNA reverse transcription, specific stem‐loop primers were designed and synthesized to target mature miR‐143‐3p. Reverse transcription was carried out using the miRNA First‐Strand cDNA Synthesis Kit (MR201‐01, Vazyme Biotech, China) under the following conditions: 25°C for 5 min, 50°C for 15 min, and 85°C for 5 min. Fluorescence quantitative PCR was performed using a fluorescence PCR instrument (CFX Connect Real‐Time PCR Detection System, Bio‐Rad Laboratories (Shanghai) Co. Ltd.). The reaction steps were as follows: predenaturation at 95°C for 10 min; 40 cycles of denaturation at 95°C for 10 s, annealing at 58°C for 30 s, and extension at 72°C for 30 s. β‐actin and U6 were used as the internal controls. The relative expression level of genes was calculated using the 2‐^△△^Ct method. The primer sequences were shown in Table 2.

**TABLE 2:** Primers used in this study.

Name	Sequences(5′‐3′)
*β‐Actin F*	TGGCACCCAGCACAATGAA
*β‐Actin R*	CTAAGTCATAGTCCGCCTAGAAGCA
*hsa_circRNA_0101125 F*	CCCCAAGGACATTCGGAGAT
*hsa_circRNA_0101125 R*	GCTGCCATAACAAAGTACCCA
*ZEB1 F*	TTTCCCATTCTGGCTCCTA
*ZEB1 R*	TCTATCTTTTGCCGTATCTGTG
*Znf148 F*	ATCGAAGTATGCCTCACCAGG
*Znf148 R*	CCTGCTTTACGCTTATAGGGACA
*ZHX3 F*	CATGTGGTTGTGGAGCAGAG
*ZHX3 R*	CTGGCTAGGGACATTCTCCTT
*U6 F*	CTCGCTTCGGCAGCACA
*U6 R*	AACGCTTCACGAATTTGCGT
*miR‐143‐3p F*	CGCGTGAGATGAAGCACTG
*miR‐143‐3p R*	AGTGCAGGGTCCGAGGTATT

Abbreviations: CircRNA, circular RNA; miR, microRNA; ZEB1, zinc finger E‐box binding homeobox 1; ZNF148, zinc finger protein 148; ZHX3, zinc fingers and homeoboxes 3.

### 2.9 Western Blot

Cell samples from each group were collected. The cell culture medium was discarded; RIPA cell lysate (C1053, ApplyGen, Beijing, China) was added, and the samples were placed on ice for 20 min. After centrifugation with a high‐speed centrifuge (12 000 r/min) for 10 min, the supernatant was taken and transferred to a new EP tube. BCA protein assay kit (E‐BC‐K318‐M, Elabscience, Wuhan, China) was used to detect protein concentration in cell supernatant. SDS‐PAGE was performed and the proteins were then transferred onto the PVDF membrane (IPVH00010, Millipore, Massachusetts, USA). The membrane was blocked with 3% skim milk for 1 h. After incubation with Rabbit Anti ZNF148 (1/1000; AF0792, Affinity, Jiangsu, China), Mouse Anti Vimentin (1/1000; 60 330–1‐Ig, Proteintech, Wuhan, China), Rabbit Anti E‐Cadherin (1/1000; AF0131, Affinity, Jiangsu, China) or Mouse Anti‐β‐Actin (1/2000; HC201, TransGen Biotech, Beijing, China) overnight, the membrane was washed three times. HRP conjugated Goat Anti Mouse IgG (H + L) (1/2000; GB23301, Servicebio, Wuhan, China) or HRP conjugated Goat Anti‐Rabbit IgG (H + L) (GB23303, Servicebio, Wuhan, China) was added to incubate the membrane for 2 h, and then the membrane was washed three times. The PVDF membrane was soaked with luminescent liquid (RJ239676, Thermo Fisher, Massachusetts, USA) and images were developed under the ultra‐high sensitivity chemiluminescence imaging system (Chemi DocTM XRS+, Bio‐Rad Laboratories (Shanghai) Co. Ltd.). Band intensity was quantified using ImageJ software (NIH, USA). Relative protein expression levels were normalized to GAPDH and expressed as fold change over control. All blots were repeated in at least three independent experiments.

### 2.10 Statistical Analysis

SPSS 20.0 software was used for statistical analysis. All experiments were repeated three times, and the quantitative results were expressed as mean ± standard deviation (X ± S). The quantitative values between the two groups were compared using an independent sample *t*‐test. The quantitative values between multiple groups were compared using one‐way ANOVA, and the pairwise comparison was conducted using the S‐N‐K method. *p* < 0.05 indicates significant differences.

## 3 Results

### 3.1 The Localization of hsa_circRNA_0101125 and the Expression of Related Genes in KYSE150 and ECA109 Cells

**FIGURE 1:**
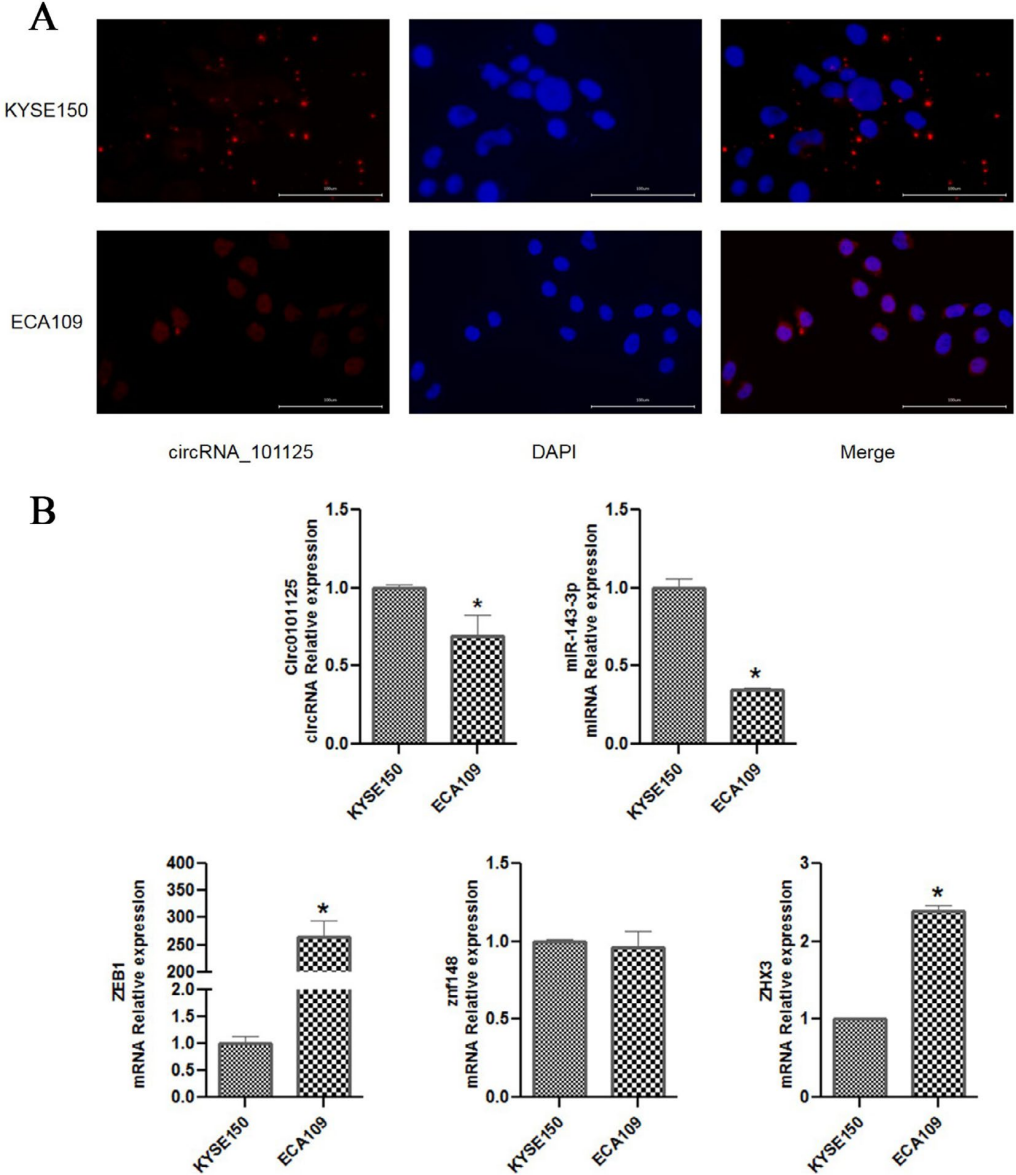
The localization of hsa_circRNA_0101125 and the expression of related genes in KYSE150 and ECA109 cells. (A) Fluorescence in situ hybridization (FISH) showing the subcellular localization of hsa_circRNA_0101125 in KYSE150 and ECA109 cells (Magnification, ×400). (B) RT‐qPCR analysis of hsa_circRNA_0101125, miR‐143‐3p, ZEB1, ZHX3, and ZNF148 expression levels in KYSE150 and ECA109 cells. *p < 0.05 compared with KYSE150. *n* = 3. CircRNA, circular RNA; miR, microRNA; ZEB1, zinc finger E‐box binding homeobox 1; ZNF148, zinc finger protein 148; ZHX3, zinc fingers and homeoboxes 3.

The FISH method was used to detect the localization of hsa_circRNA_0101125 in KYSE150 cells. As shown in Figure 1A, the hsa_circRNA_0101125 gene was located in the cytoplasm.

As shown in Figure 1B, compared with KYSE150 cells, the expression levels of hsa_circRNA_0101125 and miR‐143‐3p were lower in ECA109 cells (both *p* < 0.05). The mRNA expression levels of ZEB1 and ZHX3 were significantly increased in ECA109 cells compared with KYSE150 cells (both *p* < 0.05), while the mRNA level of ZNF148 did not differ between the two cell lines (*p* > 0.05). Although ZNF148 expression showed no significant difference between KYSE150 (derived from the cervical esophagus) and ECA109 (derived from the mid‐esophagus) cells, ZHX3 and ZEB1 were more highly expressed in the mid‐esophageal ECA109 line. Given previous reports indicating that ZNF148 is consistently upregulated in ESCC tumor tissues and independently associated with advanced stage and poor prognosis [19], we speculated that ZNF148 may exert a more universal tumor‐promoting effect across ESCC subtypes and thus selected it for further mechanistic investigation.

### 3.2 Effect of hsa_circRNA_0101125 Interference on Esophageal Cancer Cells

**FIGURE 2:**
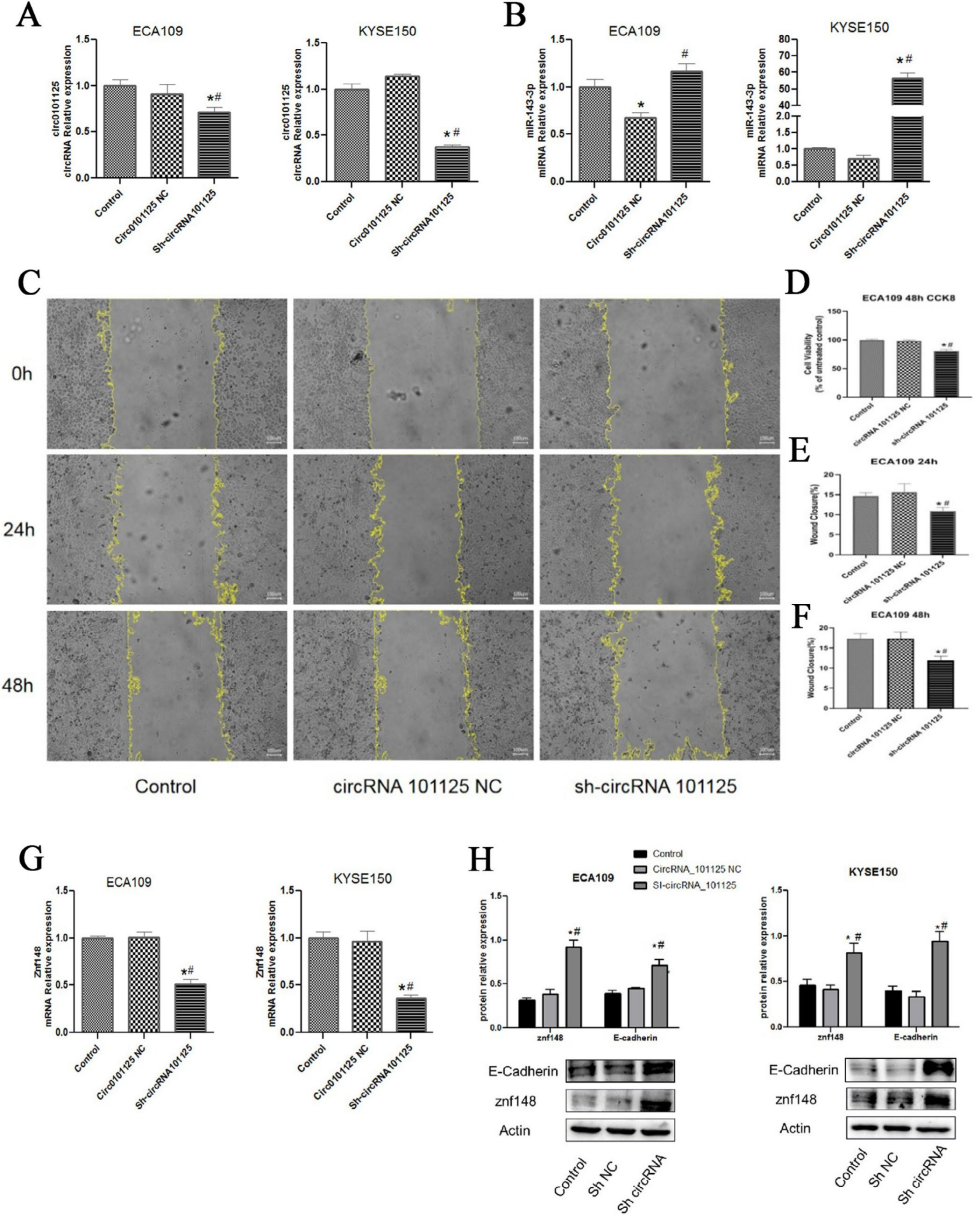
Effect of hsa_circRNA_0101125 knockdown on proliferation, migration, and epithelial marker expression in ESCC cells. (A) RT‐qPCR analysis showing the reduction of hsa_circRNA_0101125 expression in KYSE150 and ECA109 cells after transfection with sh‐hsa_circRNA_0101125. (B) RT‐qPCR analysis of miR‐143‐3p expression following hsa_circRNA_0101125 knockdown. (C) Representative images of scratch wound healing assay in ECA109 cells at 0 h and 24 h. (D‐E) Quantitative analysis of wound closure. (F) Cell viability of ECA109 cells measured by CCK‐8 assay at 24 h and 48 h after knockdown. (G) Western blot analysis of E‐cadherin protein expression in KYSE150 and ECA109 cells after knockdown. Relative expression values represent the gray intensity of E‐cadherin normalized to β‐Actin, based on densitometry analysis using ImageJ software. *p < 0.05 compared with Control, ^#^ *p* < 0.05 compared with NC. CircRNA, circular RNA; miR, microRNA.

As shown in Figure 2A, the expression of hsa_circRNA_0101125 in KYSE150 and ECA109 cells was significantly decreased after transfection of sh‐hsa_circRNA_0101125 (both *p* < 0.05), indicating that the sh‐hsa_circRNA_0101125 vector could successfully interfere with the expression of hsa_circRNA_0101125 in KYSE150 and ECA109 cells.

As shown in Figure 2B, the expression level of the miR‐143‐3p gene was significantly increased in KYSE150 and ECA109 cells after hsa_circRNA_0101125 inhibition (both *p* < 0.05).

As shown in Figure 2C–F, compared with the hsa_circRNA_0101125 NC group, the cell viability and scratch healing rate in KYSE150 and ECA109 cells were significantly decreased after hsa_circRNA_0101125 interference for 24 to 48 h (both *p* < 0.05). Specifically, the cell viability in the sh‐hsa_circRNA_0101125 group was reduced to approximately 76% of that in the control group. This suggested that hsa_circRNA_0101125 can increase the viability and migration of ESCC cells.

As shown in Figure 2G,H, the expression level of E‐Cadherin and Znf148 proteins was significantly increased in KYSE150 and ECA109 cells after hsa_circRNA_0101125 inhibition (both *p* < 0.05).

### 3.3 Results of Dual‐Luciferase Reporter Assay

**FIGURE 3:**
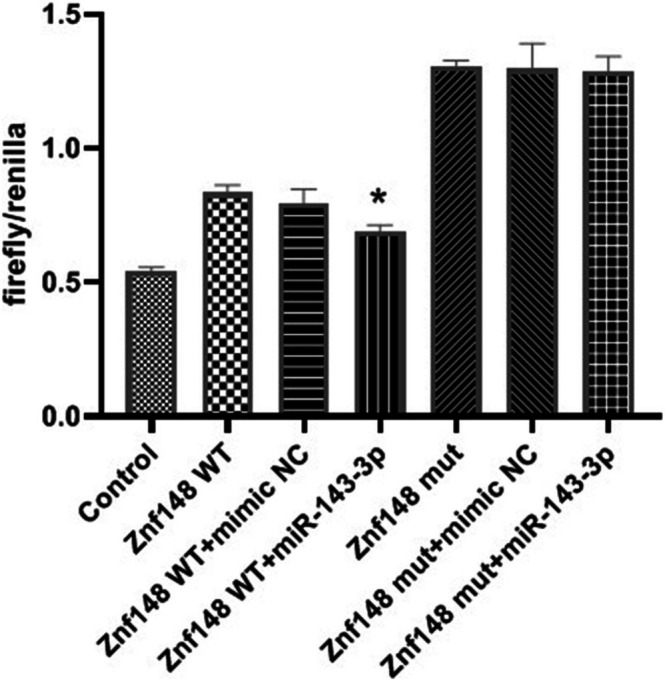
Results of dual‐luciferase reporter assay. Wild‐type (WT) and mutant (MUT) ZNF148 3′UTR sequences were cloned into the pmirGLO vector and co‐transfected with miR‐143‐3p mimic or negative control into ESCC cells. Luciferase activity was measured 48 h after transfection using the Dual‐Luciferase Reporter Assay System. The Control group refers to cells transfected without reporter plasmids or miRNA mimics, serving as a background reference. *p < 0.05 compared with ZNF148 WT + mimic NC. ZNF148, zinc finger protein 148; WT, wild type; mut, mutant.

As shown in Figure 3, compared with the Znf148 WT + mimic NC group, the luciferase/Renilla ratio in the Znf148 WT + miR‐143‐3p group decreased significantly (*p* < 0.05). The luciferase activity was significantly decreased when miR‐143‐3p was combined with ZNF148, indicating that miR‐143‐3p has a binding site with ZNF148.

### 3.4 Downregulation of miR‐143‐3p Reversed the Effect of hsa_circRNA_0101125 on KYSE150 and ECA109 Cells

**FIGURE 4:**
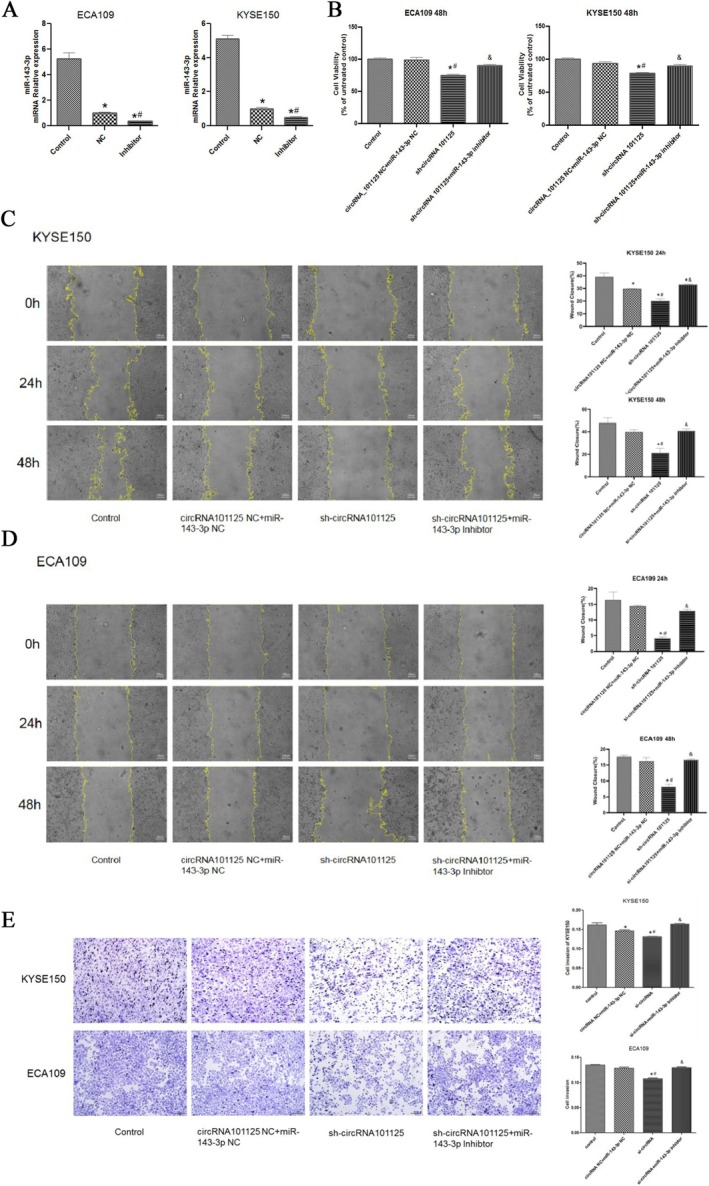
Downregulation of miR‐143‐3p reversed the effect of hsa_circRNA_0101125 on KYSE150 and ECA109 cells. (A) RT‐qPCR analysis of miR‐143‐3p expression after co‐transfection with miR‐143‐3p inhibitor and sh‐hsa_circRNA_0101125. (B) Cell viability detected by CCK‐8 assay at 24 h and 48 h after co‐transfection with miR‐143‐3p inhibitor and sh‐hsa_circRNA_0101125. (C‐D) Wound healing assay to assess cell migration after co‐transfection with miR‐143‐3p inhibitor and sh‐hsa_circRNA_0101125 (Magnification, ×400). (E) Transwell invasion assay to assess invasive ability after co‐transfection with miR‐143‐3p inhibitor and sh‐hsa_circRNA_0101125 magnification, ×400. *p < 0.05 compared with control, ^#^ *p* < 0.05 compared with NC, ^&^ *p* < 0.05 compared with shRNA. CircRNA, circular RNA; miR, microRNA.

As shown in Figure 4A, compared with the control and NC groups, the expression of miR‐143‐3p in the inhibitor group was significantly decreased in both KYSE150 and ECA109 cells (*p* < 0.05), indicating successful miR‐143‐3p interference.

As shown in Figure 4B, compared with the control and NC groups, the cell viability of the sh‐hsa_circRNA_0101125 group was significantly decreased. Compared with the sh‐hsa_circRNA_0101125 group, the sh‐hsa_circRNA_0101125 + miR‐143‐3p inhibitor group showed a significant increase in cell viability (*p* < 0.05).

As shown in Figure 4C,D, compared with the control group and NC group, cell migration ability in the sh‐hsa_circRNA_0101125 group was decreased significantly (*p* < 0.05). Compared with the sh‐hsa_circRNA_0101125 group, cell migration ability in the sh‐hsa_circRNA_0101125 + miR‐143‐3p inhibitor group was significantly increased (*p* < 0.05).

As shown in Figure 4E, compared with the control group and NC group, cell invasion ability in the sh‐hsa_circRNA_0101125 group was decreased significantly (*p* < 0.05). Compared with the sh‐hsa_circRNA_0101125 group, cell invasion ability in the sh‐hsa_circRNA_0101125 + miR‐143‐3p inhibitor group was significantly increased (*p* < 0.05).

### 3.5 Downregulation of miR‐143‐3p Reversed the Effect of hsa_circRNA_0101125 on Related Proteins in KYSE150 and ECA109 Cells

**FIGURE 5:**
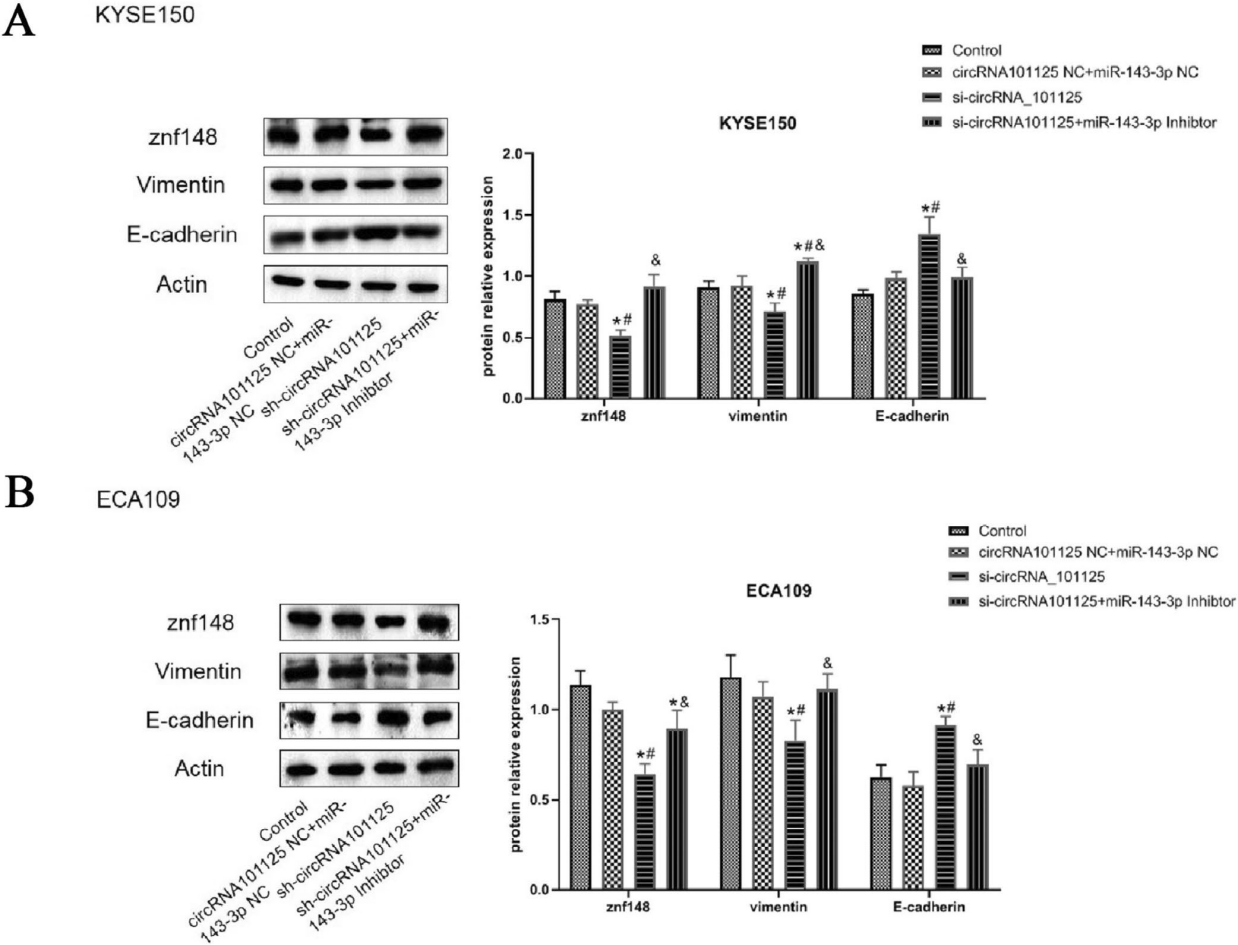
Western blot analysis of EMT‐related proteins in KYSE150 and ECA109 cells following hsa_circRNA_0101125 knockdown and miR‐143‐3p inhibition. Protein levels of ZNF148, Vimentin, and E‐cadherin were evaluated by Western blotting in the control, NC, sh‐hsa_circRNA_0101125, and sh‐hsa_circRNA_0101125 + miR‐143‐3p inhibitor groups. Relative expression values represent the gray intensity of E‐cadherin normalized to β‐Actin, based on densitometry analysis using ImageJ software. *p < 0.05 compared with control; ^#^ *p* < 0.05 compared with NC, ^&^ *p* < 0.05 compared with shRNA. CircRNA, circular RNA; miR, microRNA; ZNF148, zinc finger protein.

As shown in Figure 5A,B, compared with the control group and NC group, the expression of ZNF148 and Vimentin in the sh‐hsa_circRNA_0101125 group was decreased significantly. Compared with the sh‐hsa_circRNA_0101125 group, the expression of ZNF148 and Vimentin in the sh‐hsa_circRNA_0101125 + miR‐143‐3p inhibitor group was significantly increased (*p* < 0.05).

Compared with the control group and NC group, the expression of E‐cadherin in the sh‐hsa_circRNA_0101125 group was increased significantly. Compared with the sh‐hsa_circRNA_0101125 group, the expression of E‐cadherin in the sh‐hsa_circRNA_0101125 + miR‐143‐3p inhibitor group was significantly decreased (*p* < 0.05).

## 4 Discussion

ESCC is a cancer with high mortality and poor prognosis. ESCC cells have a strong ability to proliferate and migrate, leading to cancer metastasis [3, 4, 21]. There have been some reports on the mechanisms underlying the regulatory role of circRNAs in ESCC, and circRNAs can regulate the proliferation and migration ability of cancer cells through ceRNA mechanisms [6, 21]. Our previous study found that hsa_circRNA_0101125 is highly expressed in ESCC tissue, but the regulatory mechanism of hsa_circRNA_0101125 in cancer is currently unclear [7]. In this study, we demonstrated that hsa_circRNA_0101125 promotes proliferation, migration, and invasion of ESCC cells by acting as a ceRNA to sponge miR‐143‐3p, thereby upregulating the expression of its downstream target ZNF148. To evaluate whether this regulatory axis is broadly involved across different ESCC subtypes, we employed two representative cell lines: KYSE150 (derived from the cervical esophagus) and ECA109 (derived from the mid‐esophagus). Consistently, hsa_circRNA_0101125 was found to be highly expressed in both cell lines, while miR‐143‐3p exhibited an inverse expression pattern. In particular, we observed that KYSE150 cells showed relatively higher expression of hsa_circRNA_0101125 and lower levels of miR‐143‐3p compared to ECA109, suggesting subtype‐specific differences. The differential expression of hsa_circRNA_0101125, miR‐143‐3p, and ZNF148 between the two cell lines implies that while this axis is functionally relevant in ESCC, its regulatory strength or expression dynamics may vary with tumor location. These findings might provide a basis for exploring whether this axis plays a universal or location‐dependent role in ESCC progression and could guide future efforts in developing broadly applicable therapeutic strategies.

MiRNAs are single stranded RNAs that contain 20–23 non‐coding nucleotides and play important roles in regulating cancer development. MiR‐143‐3p, as a tumor suppressor, can inhibit the occurrence of cancers such as ESCC, ovarian cancer, and gallbladder cancer [11, 22, 23]. ZNF148, also known as ZBP89, ZBP‐89, and ZFP148, is a multifunctional zinc finger transcription factor that plays an important regulatory role in cell growth, apoptosis, and carcinogenesis [24]. There are many controversies regarding the role of ZNF148 in regulating tumor development. ZNF148 can inhibit the stemness transformation of liver cancer cells [25, 26, 27]; however, ZNF148 knockdown can inhibit the growth of colon cancer [28]. Cheng et al. found that ZNF148 promotes the stemness transformation of glioma cells by upregulating PTX3 [29]. In this study, the interaction between miR‐143‐3p and ZNF148 was verified by dual‐luciferase reporter assay. After interference with hsa_circRNA_0101125, the expression level of miR‐143‐3p was increased, and the expression level of ZNF148 protein was decreased. In the meanwhile, the migration ability and the viability of ECA109 cells were significantly decreased. These results suggested that hsa_circRNA_0101125 is an oncogenic gene in the development of ESCC, which can inhibit the expression of miR‐143‐3p through the ceRNA mechanism, thereby activating ZNF148 and promoting the proliferation and migration of ESCC cells.

Epithelial‐mesenchymal transition (EMT) is a cellular biological process in which epithelial cells gradually lose their cellular characteristics and morphology and gradually acquire the characteristics of mesenchymal phenotype [30]. In the process of epithelial cells transforming into mesenchymal cells, the expression of E‐cadherin, cytokeratins, and claudins in epithelial cells was decreased, while the expression of N‐cadherin, fibronectin, and Vimentin in mesenchymal cells was increased [31]. Cancer stem cells (CSCs) are cancer cells with the characteristics of normal stem cells that possess the ability to self‐renew and can replicate infinitely [32]. The drug resistance and tumorigenicity of cancer cells were increased following stemness transformation [33]. Cancer cells can obtain the characteristics of stem cells through EMT. The changes in the expression of EMT‐related proteins Vimentin, E‐cadherin, and N‐cadherin have been observed in ESCC cells during stemness transformation [34, 35]. Meanwhile, many tumors originating from epithelial cells require EMT to undergo invasion and metastasis [12]. Therefore, EMT inhibition in cancer cells is an important treatment for cancer. Lee et al. found that Brucea javanica bitter alcohol can inhibit the proliferation and migration of liver cancer cells by inhibiting EMT through STAT3 [36]. In this study, after interference with hsa_circRNA_0101125, the expression of epithelial cell characteristic protein E‐cadherin was increased, and the proliferation and migration ability of cells was decreased. This finding indicates that interference with hsa_circRNA_0101125 inhibits EMT, thereby inhibiting the stemness transformation of ESCC. To further verify the relationship between hsa_circRNA_0101125 and miR‐143‐3p, the expression of miR‐143‐3p was suppressed in cells interfered with hsa_circRNA_0101125. The results showed that after miR‐143‐3p inhibition, the inhibitory effect of hsa_circRNA_0101125 interference on the proliferation and migration of ESCC cells was weakened. Compared with the hsa_circRNA_0101125 interference group, the expression levels of ZNF148 protein and Vimentin protein were increased, while the expression of E‐cadherin was decreased. ZNF148 may activate the EMT pathway through the miR‐143/ZNF148 axis, thereby promoting cell invasion. However, the regulatory mechanism is still unclear, and it requires further exploration.

Despite the significant findings, several limitations should be acknowledged in this study. First, although our study demonstrated that hsa_circRNA_0101125 regulates ZNF148 expression through sponging miR‐143‐3p, the regulatory effect of miR‐143‐3p on ZNF148 protein expression was assessed in the context of hsa_circRNA_0101125 knockdown and rescue. This approach provided indirect evidence supporting the negative regulatory relationship between miR‐143‐3p and ZNF148, as reflected by the downregulation of ZNF148 upon hsa_circRNA_0101125 silencing and its partial restoration following miR‐143‐3p inhibition. However, direct validation of miR‐143‐3p's impact on ZNF148 protein levels in isolation was not performed in this study. Future investigations might explore this regulatory interaction more thoroughly by independently evaluating the effects of miR‐143‐3p mimics and inhibitors on ZNF148 expression. Second, the observed changes in E‐cadherin and Vimentin expression following modulation of the hsa_circRNA_0101125/miR‐143‐3p axis suggest a potential link to EMT. While miR‐143‐3p has been previously associated with EMT regulation [37], the specific role of ZNF148 in modulating EMT markers remains unclear. Future studies are warranted to elucidate whether ZNF148 directly regulates E‐cadherin or Vimentin expression, or acts through intermediary EMT‐related pathways. Third, we observed a decrease in miR‐143‐3p expression in the NC groups of sh‐circRNA_101125 or miR‐143‐3p‐inhibitor, particularly in ECA109 cells. This may reflect nonspecific cellular responses to transfection reagents or potential transient off‐target effects from the non‐targeting shRNA construct. Although the statistically significant changes in miR‐143‐3p expression in the experimental group support our findings, this observation highlights the need for additional validation using alternative transfection methods or delivery systems in future work. Future studies might further investigate the molecular basis of such nonspecific miRNA fluctuations, especially in transfection‐sensitive cell lines like ECA109. Employing multiple negative control strategies and optimizing transfection conditions could improve the reproducibility and physiological relevance of circRNA/miRNA functional studies.

## 5 Conclusions

In conclusion, this study revealed that hsa_circRNA_0101125, as a tumor promoting factor, plays an important role in the carcinogenesis of ESCC. The hsa_circRNA_0101125 regulates the proliferation, migration, and stemness transformation of ESCC cells through the miR‐143‐3p/ZNF148 axis. This study may provide a potential new treatment strategy for ESCC.

## Author Contributions

**Hao Chen:** conceptualization, investigation, methodology, data curation, formal analysis, – writing – original draft. **Songtao Xue**, **Shiqiang Cao**, **Jiarong Zhang**, **Yuanpu Wei:** investigation, methodology, data curation, formal analysis, software. **Chun Chen**, **Guobing Xu:** conceptualization, project administration, supervision, writing – review and editing.

## Disclosure

The authors have nothing to report.

## Ethics Statement

This research did not involve human or animal studies, and therefore ethics committee approval is not applicable.

## Consent

The authors have nothing to report.

## Conflicts of Interest

The authors declare no conflicts of interest.

## Data Availability

The dataset generated or analyzed in this study can be provided upon reasonable request.
